# Comprehensive growth index monitoring of desert steppe grassland vegetation based on UAV hyperspectral

**DOI:** 10.3389/fpls.2022.1050999

**Published:** 2023-01-24

**Authors:** Xiaomin Liu, Haichao Wang, Yanwei Cao, Yaotian Yang, Xiaotian Sun, Kai Sun, Ying Li, Junyao Zhang, Zhiyong Pei

**Affiliations:** ^1^ Inner Mongolia Agricultural University, Hohhot, China; ^2^ Inner Mongolia Yellow River Ecological Research Institute, Hohhot, China; ^3^ Collaborative Innovation Center for Integrated Management of Water Resources and Water Environment in Inner Mongolia Section of Yellow River Basin, Hohhot, China; ^4^ Ordos City Science and Technology Business Development Center, Ordos, China

**Keywords:** comprehensive growth index, spectral analysis, UAV, desert steppe, gradation

## Abstract

The goal of this study was to establish a comprehensive growth index (CGI) of grassland vegetation for monitor the overall condition of the grassland. Taking the desert grassland in Otuoke Banner, Ordos City, Inner Mongolia as the research object, this study integrates five indicators. First, the optimal band of the unmanned aerial vehicle hyperspectral data is optimized using the correlation analysis, successive projection algorithm (SPA), optimum index factor method, and band combination index method. A dual-band spectral index in good correlation with the CGI is then constructed in the optimal band. Afterwards, a CGI characterization model is established in accordance with the partial least squares regression (PLSR) algorithm and its accuracy is analyzed. Finally, the CGI of the study area is estimated. The experimental results are as follows. 1) The R^2^ of models built using the training samples of the spectral indices corresponding to the optimal spectra screened by the SPA method was 0.7835, RMSE was 0.0712, and RE was 6.89%, less than 10%. The R^2^ of the Validation samples was 0.7698, RMSE was 0.0471, and RE was 6.36%, less than 10%, highest precision. 2) Models were built using the spectral indices corresponding to the optimal spectra screened by the SPA method, and the CGI mean values were inverted. A comparison of the mean measured CGI values of the sample quadrat of the test area showed that the mean relative error was 3.82%. The results show that the vegetation growth of desert-steppe grasslands can be adequately monitored, providing technical support for the rapid and accurate diagnosis of grassland conditions. However, there are still shortcomings in this study. 1) The research area for this study was mainly in the desert steppe in Otuoke Banner, Ordos, hence the relevance and universality of the findings need to be verified, and subsequent experiments need to be carried out on desert steppes in other regions or even other types of grasslands to test the universality of the model. 2) In this study, the influence of soil background and litter on the spectral reflectance is not considered in depth. In addition, the influence of sensor observation angle and solar elevation angle on the inversion model demands further investigation efforts.

## 1 Introduction

Desert steppe is a terrestrial ecosystem that transitions from grassland to desert in central Asia and is located in the ecotone of grassland and desert. Accounting for 34.7% of the total grassland area in Northern China, it is an important part of the grassland in Inner Mongolia, as well as an important production base for animal husbandry in Northern China. The latest China Forestry and Grassland Development Report states that 50% to 60% of China’s natural grasslands are degraded to varying degrees, and desert grasslands are fragile ecosystems more prone to degradation than other types of grasslands. Therefore, the accurate and efficient identification of desert grassland conditions is important research content for grassland ecological restoration and sustainable development.

The key to identifying grassland conditions is to obtain information about the growth and distribution of the grassland vegetation rapidly and accurately. The traditional approach mainly involves field sampling and laboratory test analyses, which can be complicated, tedious, and time-consuming and seldom meet the needs of regional dynamic monitoring.

Unmanned aerial vehicle (UAV) hyperspectral remote sensing has advantages such as high temporal and spatial resolutions, timely data acquisition, operational convenience, high mobility, low cloud interference, and low cost. Hence, the method has been widely used in vegetation index monitoring in recent years (Fu et al., 2021). The introduction of UAV hyperspectral remote sensing technology into the rapid estimation of grassland vegetation growth has significant research and practical value. UAV hyperspectral remote sensing focuses on indicators such as fractional vegetation cover (FVC), above ground biomass (AGB), and leaf area index (LAI).

First, vegetation cover, which is the ratio of the vertical projection area of vegetation to the total land area, is an important indicator for measuring surface vegetation condition. Yue et al. (2021) proposed a fan-shaped method (FSM) using canopy chlorophyll content (CCC) and spectral index (SI). They created a two-dimensional scatter plot using the FSM, nonlinear regression, and a pixel dichotomy model (PDM) to calculate the inversion of soybean cover, thereby achieving accurate cover estimation. Abdelbaki et al. (2021) used UAV hyperspectral remote sensing to estimate the LAI, FVC, and CCC in six stages of the potato growth season and precision of the model is analyzed. Feng et al. (2017) proposed a vegetation cover estimation method based on UAV hyperspectral data. They used the red edge slope *k* as the parameter, as well as mixed pixel decomposition, geometric correction, supervised classification, and the PDM. However, the method considerably influences the red edge slope when the spectrum of the red edge interval contains a measurement error, resulting in insufficient stability. Tang et al. (2020) examined the responses of fractional vegetation cover (FVC) and normalized difference vegetation index (NDVI) to hydrothermal gradient in arid desert areas using unmanned aerial vehicle (UAV) remote sensing. FVC of each sampling point was obtained through unmanned aerial vehicle remote sensing (FVCU), which was used to examine the FVC that was retrieved by the pixel binary model (FVCM). FVCM reflected the vegetation coverage of Alxa region with an accuracy of 83.1%, which were 14.8% lower than the real value. Ge et al. (2017) proposed theMODIS NDVI and EVI data from 41 field measurements in the eastern headwaters of the Yellow River were used. In combination with the alpine grassland coverage data obtained by an agricultural digital camera (ADC), ordinary digital camera (i. e., Canon 70D) and UAV images, grassland coverage inversion models were constructed using MODIS vegetation indices. The optimal remote sensing model was used to analyze the grassland coverage dynamics from 2000 to 2015. The results indicated that, Compared with the grassland coverage calculated with the Canon, images from the ADC and UAV under 30 m and 100 m flight height with the two MODIS vegetation indices respectively, the MODIS NDVI was more sensitive to grassland vegetation coverage retrieved by UAV under 30 m flight height, the optimal model was y=65. 41321n(NDVI)+109. 1763 (R2 = 0. 7575, RMSEP=8. 4780). Wei et al. (2021) used UAV-based hyperspectral data to compare the accuracy of three estimation models—the dimidiate pixel, Carlson, and Baret models—as well as the linear mixed model, which is currently a commonly used model, polynomial post-nonlinear mixing model, and normal compositional model (NCM), considering spectral variation. The results showed that the NCM model achieved the optimal estimation. Liu et al. (2021) evaluated the sensitivity and estimation accuracy of the NDVI of grassland cover by analyzing the artificial grassland hyperspectral images obtained by the air-borne Resonon Pika XC2 hyperspectral imaging camera. They found that the narrow-band NDVI of the four types of mainstream satellite images had higher inversion accuracy for grassland cover, whereas the wide-band inversion accuracy was attenuated to a certain degree.

Furthermore, AGB is the organic matter content of the vegetation above the soil per unit area and usually refers to the dry weight of the stems, branches, foliage, flowers, and fruits. AGB is a key biophysical parameter that reflects vegetation growth; thus, it is used for monitoring the growth of pasture and rationalizing grazing. Yang et al. (2021) estimated the yield of a new type of winter wheat in the North China Plain using UAV hyperspectral remote sensing. They established a new type of winter wheat yield estimation model (CW-RF) using the random forest (RF) algorithm. Liang et al. (2021) applied machine learning to structural and spectral information provided by UAV hyperspectral remote sensing to estimate maize biomass. They evaluated and compared four machine learning regression algorithms (multiple linear regression, support vector machine, artificial neural network, and RF) and proposed an improved method for extracting plant height and biomass information from drone imagery, as well as a volumetric indicator, BIOVP. Kang et al. (2021) proposed an optimization method for forage canopy spectral reconstruction. The method considers both data simplification and spectral fidelity, which effectively reduces the amount of data needed and ensures accurate AGB prediction. Liu et al. (2021) Correlation analysis method (CAM), random frog method (RFM) and Gaussian process regression bands analysis tool (GPR-BAT) were used to screen canopy original spectra (COS) and first-order derivative spectra (FDS) for sensitive wavelengths, respectively, combined with partial least squares regression (PLSR) and Gaussian process regression (GPR) techniques to establish AGB estimation models for each fertility period of potatoes and the estimation effects of different models were compared.

Finally, LAI is the ratio of the total area of plant leaves in a unit land area to the land area. It is an important indicator in the characterization of vegetation photosynthesis, respiration, and transpiration, and it is the primary basis for evaluating vegetation growth and yield. Tao et al. (2020) proposed the hyperspectral sensor mounted on an unmanned aerial vehicle was used to obtain vegetation indices and red-edge parameters, and stepwise regression (SWR) and partial least squares regression (PLSR) methods were used to accurately estimate the AGB and LAI based on these vegetation indices, red-edge parameters, and their combination. The results show that, combining vegetation indices with red-edge parameters and using the PLSR method can improve the estimation of AGB and LAI. Pei et al. (2017) constructed the normalized difference spectral index (NDSI), ratio spectral index (RSI), and simple spectral index (SSI) to determine the correlation between the spectral index and wheat growth using a single band and any two bands in the UAV hyperspectral range of 450–882 nm. They used PLSR to establish an inversion model, whose results determined the overall difference in wheat growth. Sun et al. (2022) constructed a new dual-band index by screening the optimal bands of the UAV hyperspectral data for wheat through a successive projection algorithm (SPA), optimal index method (OIF), and band combination index method (BCI), respectively. They then applied support vector regression, PLSR, and RF regression (RFR) to estimate LAI. Owing to the sample quantity limitation, the model universality requires further research.

Currently, many studies have investigated the inversion of high-coverage vegetation indicators using UAV hyperspectral remote sensing, and accurate inversion models have been developed. However, relatively few studies have investigated the quantitative characterization of vegetation indexes in desert steppe with a low vegetation cover, low plant height, and high soil brightness. Most of the studies have investigated vegetation growth using single indicators, including FVC, AGB, and LAI, instead of a comprehensive index. Studies on the estimation of several growth indices have been limited to monitoring each growth parameter individually/separately and have not integrated indices that reflect vegetation growth (Hansen and Schjoerring, 2003; Tan et al., 2011; Wang et al., 2022).

Hyperspectral remote sensing data are highly suitable for monitoring grassland degradation because of their high spectral resolution and rich information. However, because of the significant amount and high band dimension of hyperspectral data, spectral information could be invalid, redundant, and overlapping, which renders full-band inversion models unstable and makes it difficult to improve the model accuracy. Therefore, it is necessary to explore methods for selecting key wavelength variables to improve model prediction performance by filtering out interfering, redundant, and co-linear information. Multiple band selection methods with good results have been developed. However, most studies currently use raw spectral reflectance or integer order differentiation for the screening and modeling of one-dimensional spectral bands, ignoring the effect of inter-band correlation. The spectral index (SI) is the most important remote sensing parameter and is obtained by a mathematical combination of several waveband data. It has better sensitivity than that of one-dimensional spectra and better eliminates the intra-band autocorrelation. Additionally, the SI effectively reduces or eliminates environmental noise, enhances the spectral feature response, and improves the modeling accuracy, making it widely recognized in applying vegetation physiochemical indices to inversion. However, the influence of existing band selection methods on the constructed vegetation index has not been comparatively analyzed, and the conventional dual-band index has problems of regionality, limitati and poor effectiveness.

**Figure 1:**
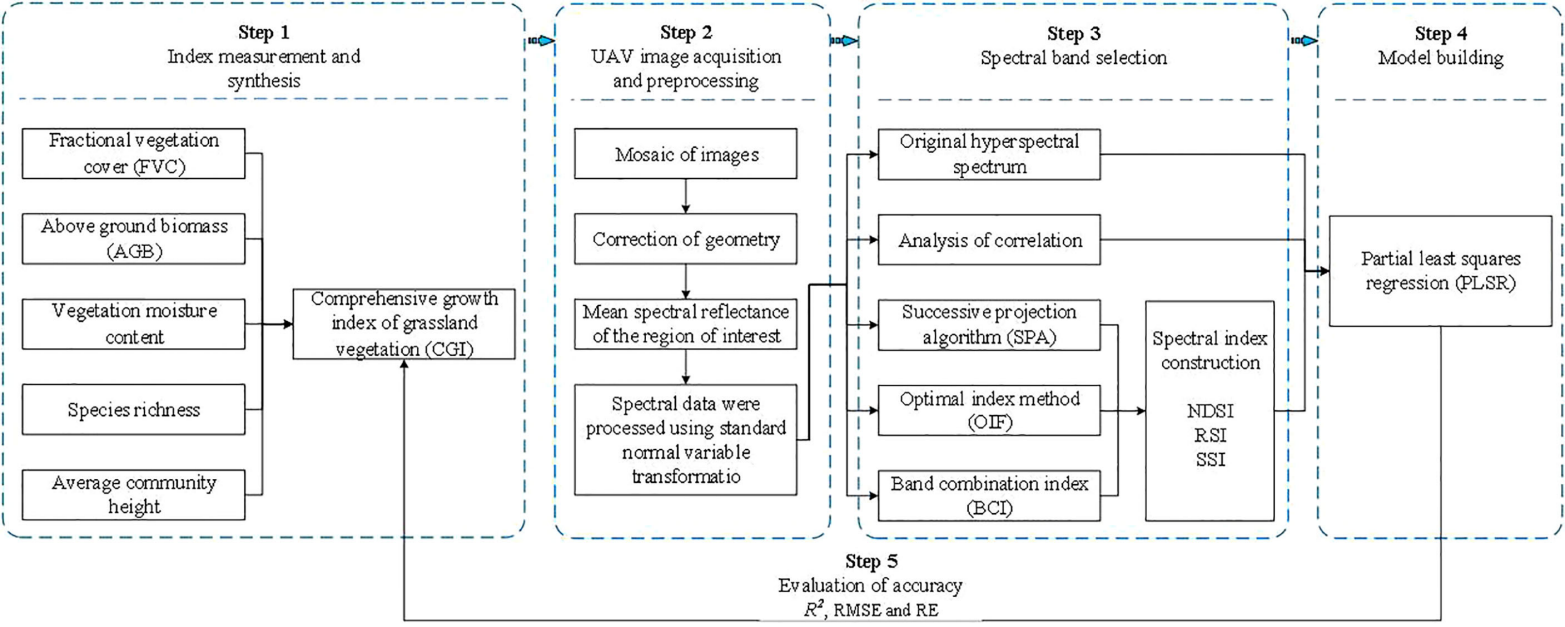
Experimental flow.

This study investigated the applicability of UAV hyperspectral data in analyzing the comprehensive growth index (CGI) characterization of desert-steppe grassland vegetation. with the aim of diagnosing grassland conditions rapidly and accurately and providing a scientific basis and technical support for scientific protection and rational utilization. First, the five indices reflecting the growth status of grassland vegetation were measured in the test area. The indices are fractional vegetation cover (FVC), above-ground biomass (AGB), vegetation moisture content (VMC), species richness (SR), and average community height (ACH). The five indices were integrated into the CGI of grassland vegetation using the equal weight method. The second step was data pre-processing, including acquisition, concatenation, and geometry correction of UAV images. The third step was the screening of the optimal pre-processed hyperspectral bands using the SPA, OIF, and BCI methods, respectively, and constructing spectral indices (NDSI, RSI, and SSI). In the fourth step, the PLSR algorithm was used to build the model of CGI characterization of grassland vegetation. The spectral index corresponding to the original spectrum, optimal spectrum screened by correlation analysis, optimal spectrum screened by the SPA method, optimal spectra screened by the OIF method, and optimal spectra screened by the BCI method were taken as input variables; the CGI of the ground vegetation was taken as the dependent variable. Finally, the results were evaluated using the coefficient of determination(*R* ^2^), root mean square error (RMSE), and relative error (RE). The flowchart of the study is presented in **Figure 1**.

## 2 Material and methods

### 2.1 Overview of the study area

**Figure 2:**
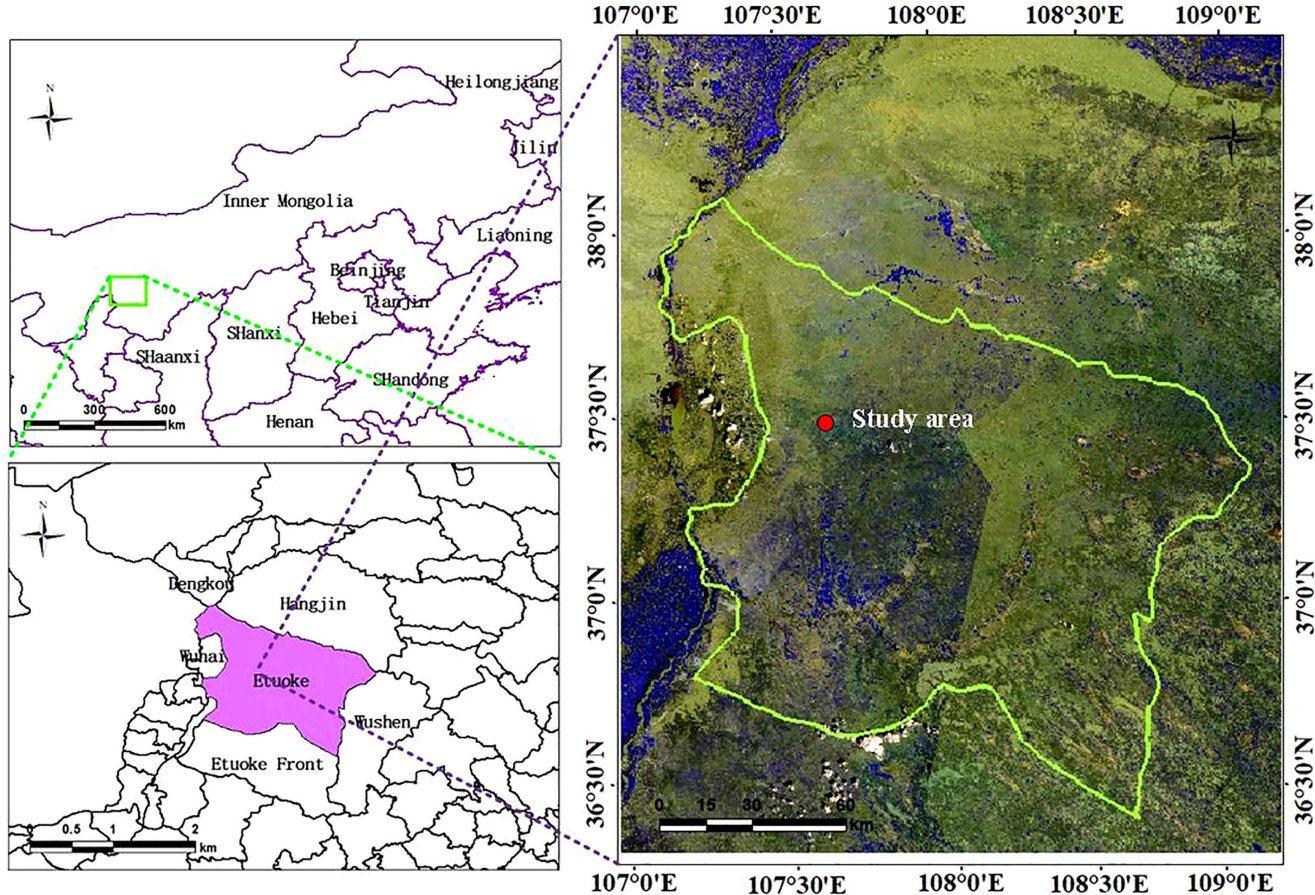
Study area.

**Figure 3:**
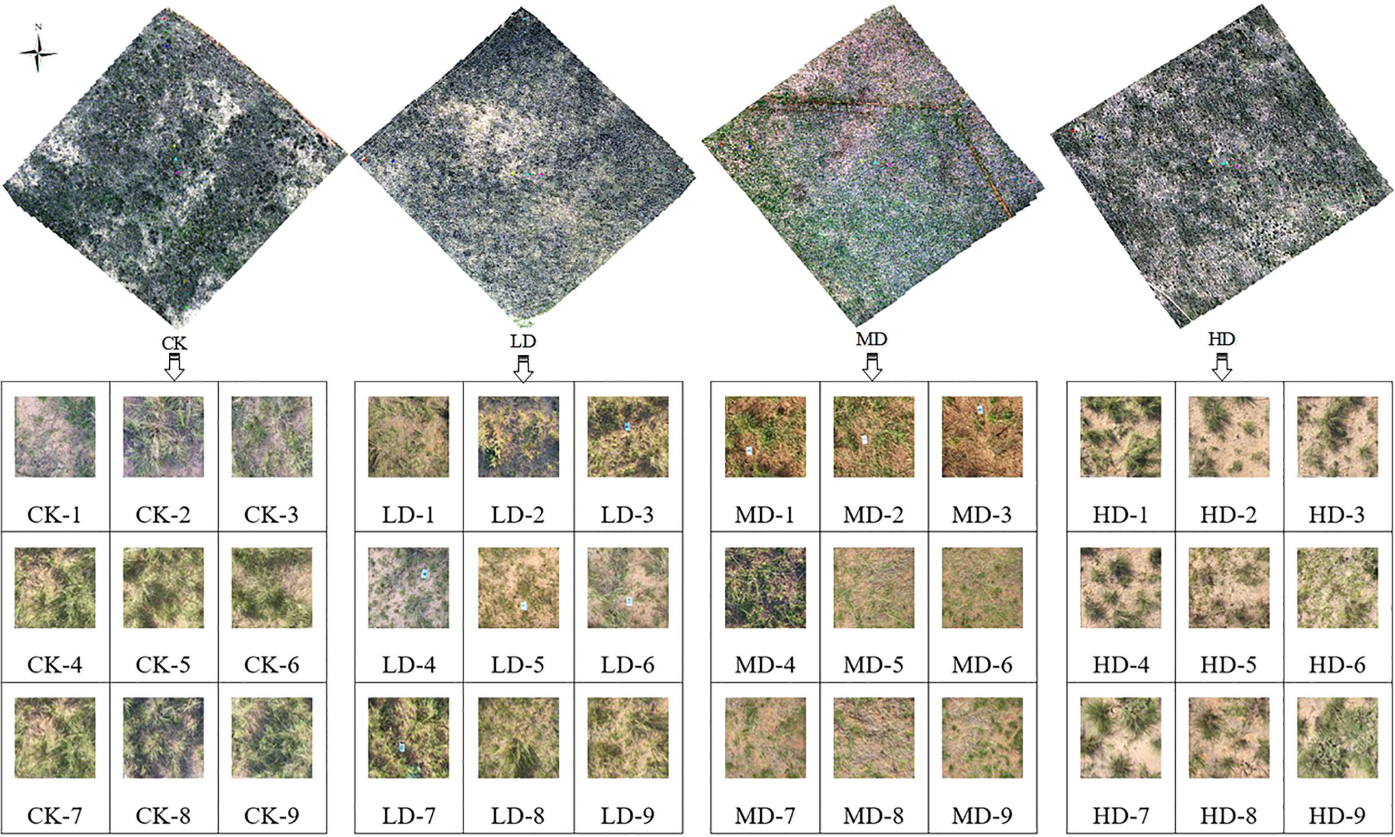
Test area and sample quadrat.

The test area, which was a sandy desert steppe subtype, was selected in the Ordos Banner of Inner Mongolia, located at a longitude of 106°41’-108°54’ east and a latitude of 38°18’-40°11’ north. The schematic of the study area is shown in **Figure 2**. With an average annual precipitation of 267 mm and an evaporation of 2480 mm (more than 9 times the average annual precipitation) from 1964 to 2021, the test area is relatively dry throughout the year. The precipitation in the growing season from April to September is 241.8 mm, accounting for 91.24% of the annual total, and the average relative humidity in the whole banner has been 48% for many years. According to the topographic map and the satellite remote sensing images of Ordos City, the monitoring data from the grassland supervision station of Etuoke Banner over the past 30 years, and the field investigation results, four groups of experimental areas with approximately the same grazing intensity and soil type (brown calcic soil) were finally selected from the 18 observation points of the monitoring station. The areas are important local pastoral areas that show obvious degradation gradients, as shown in **Figure 3**. The degradation gradient was preliminarily determined as the undegraded area (CK), light degradation area (LD), moderate degradation area (MD), and high degradation area (HD). The vegetation community in the experimental area was constructed with Stipa breviflora as the constructive species, *Caragana stenophylla* Pojark and *Cleistogenes songorica* as the dominant species, *Salsolacollina* Pall., *Echinops gmelinii* Turcz., *Carex duriuscula* C. A. Mey., *Allium mongolicum* Regel, and *Artemisia frigida* Willd. as the main associated species, and *Peganum harmala* L. as the indicator of grassland degradation in the experimental area plants, and *Oxytropis microphylla* (Pall.) DC. and *Achnatherum inebrians* (Hance) Keng as poisonous weeds (Wang et al., 2017).

The test was conducted from July 15 to July 31, 2021, with a test area of 100 m × 100 m set up in each of the four groups. In each test area, transect lines were placed along the diagonal directions, and nine quadrats of 1 × 1 m were set at equal distances and marked, where each quadrat was located using centimeter-level GPS. Fixed whiteboards were set at the four vertices for subsequent data preprocessing, as shown in **Figure 3**.

The following standards were followed: Classification standard of utilization units of natural grasslands (GB/T 34751-2017) (national standard), Parameters for degradation, sandification, and salification of rangelands (GB/19377-2003) (national standard), and Inner Mongolian standards for natural grassland degradation (DB15/T323-1999) (local standard).

### 2.2 Data collection

#### 2.2.1 Acquisition of UAV + hyperspectral data

A DJI M600 PRO was equipped with a German airborne frame-type Cubert UHD-185 Firefly (UHD-185, German Cubert, German) hyperspectral imager to acquire hyperspectral data in four study areas, Ensuring that the spectrometer lens is pointed vertically downward during the measurement, with a spectral range of 450–950 nm, an imaging speed of 5 cubes/s, a field of view (FOV) of 35.75°, a spectral resolution of 4 nm, and 126 output bands. Measurements were taken in a sunny weather with a wind force less than that of level 3, and the time of acquisition was from 10:00 to 14:00 (Beijing time). The time period was selected because it offered sufficient altitude angle and stable light conditions. Ground visibility was at least 15 km, and no cirrus or dense clouds were present. Dark current and whiteboard imaging were corrected before each take-off. For the data acquisition from the study area, the cruise height was set to 30 m, the heading overlap rate to 80%, the side overlap rate to 70%, the cruising speed to 2.4 m/s, the hyperspectral spatial resolution to approximately 1.94 cm, and the width to approximately 19.35 m.

#### 2.2.2 Ground data acquisition

The ground data were collected after the UAV hyperspectral remote sensing data acquisition.

1) Acquisition of FVC
A Canon EOS 6D (EOS 6D Mark II, Canon, Japan) digital camera was fixed on a Coman TG340CT (TG340CT, Coman, Italy) tripod and was placed 1.7 m from the 1 m × 1 m quadrats to take pictures according to the FOV. The captured images were RGB color space images, among which all quadrat canopy images were saved to a memory card for subsequent processing.
2) Species Richness Measurement
The number of species in each quadrat was counted in the field survey and indoor image discrimination.
3) Determination of Average Community Height
The individual heights of all vegetation in the quadrats under natural conditions were measured with a steel ruler, and the average height of the community in the quadrats was obtained by dividing the sum of the individual heights by the number of individuals.
4) Determination of AGB and Moisture Content Determination
The vegetation in the quadrats was cut along the ground surface, put into resealable bags after classification, and labeled. After the fresh weight was measured, the samples were brought back to the laboratory. They were baked at 65°C for more than 48 h until they reached a constant weight after 2 h of treatment at 105°C in an oven. Afterward, the dry biomass was recorded, and the community water content was calculated.

### 2.3 Data Preprocessing

#### 2.3.1 Coverage measurement

Dynamic Butterworth homomorphic filtering was used to fill in the light in the grassland vegetation images, and K-means clustering was used to segment the compensated images. Finally, the grassland vegetation cover was measured according to the definition of vegetation cover (Wang et al., 2018).

#### 2.3.2 CGI determine

The quality of regional ecology is mainly determined by the characteristic index features of vegetation. From the perspective of ecological characteristics, the ecological dynamic changes of desert steppe are mainly manifested through indicators such as FVC, SR, ACH, AGB, and VMC. Compared with using a single index, combining the above indices can reflect the ecological conditions of grasslands more accurately. Therefore, a new index, namely the CGI, was established in this study by integrating the indices reflecting the growth status of grassland vegetation. The five indicators (FVC, SR, ACH, AGB, and VMC) of the 36 quadrats were normalized using Equation 1 to prevent the data being in different orders of magnitude and units from influencing the results. Subsequently, the equal weight method (Equation 2) was used, with the weight of the indicators set to 0.2, to integrate the five indicators into a new index, namely the CGI, where 36 CGIs were obtained in the 36 quadrats.


(1) $$X_{i}^{*}=X_{i}/\max(X_{i})$$

(2) $$\text{CGI}=\frac{1}{5}\times \sum_{i=1}^{5}X_{i}^{*}$$

where *i* is the indicator type (*i*=1, 2, 3, 4, 5)); $X_{i}^{*}$ is the normalized value of the *i*th class index; *X_i_* is the original value of the *i*th class indicator; and max(*Xi*) is the maximum of the original *i*th class indicator.

#### 2.3.3 UAV hyperspectral data preprocessing

(1) Concatenation and Geometry Correction

First, the hyperspectral images were concatenated using the Agisoft PhotoScan software according to the location information. The panoramic hyperspectral images were exported in TIFF format using the Cuber-Pi-lot software (Cuber, Germany). The concatenated hyperspectral images were radiometrically corrected using the SpecView software to eliminate systematic and random radiometric distortion or aberration generated during data acquisition and transmission (Yang et al., 2019). The reflectance was then corrected to convert the digital DN values of the radiometrically corrected hyperspectral images into reflectance values, which is expressed using the following equation.


(3) $$R_{\text{ref}}=\frac{{\text{DN}}_{\text{raw}}-{\text{DN}}_{\text{dark}}}{{\text{DN}}_{\text{white}}-{\text{DN}}_{\text{dark}}}$$

Where *R_ref_* denotes the image reflectance value corrected by reflectance, DN_raw_ is the DN value of the original image, DN_dark_ is the internal systematic error generated during the hyperspectral imager measurement, and DN_white_ is the white board data measured by the camera.

(2) Mean Spectral Reflectance of the Region of Interest

Further, the vectors of the test and sample areas were divided on the geometrically corrected hyperspectral images using the ArcGIS software, and the vector files and corresponding sample quadrat names were numbered. Then the average spectral reflectance of the area of interest was extracted using the interactive data language program, and the average spectral reflectance was used as the spectral reflectance of the canopy in different areas. After that, the spectral reflectance of the 36 quadrats and 4 test areas was drawn by differentiating the quadratic and test areas in the reflectance images. Finally, in order to reduce interference such as noise and spectral line drift, the spectral data were processed using standard normal variable transformation (SNV) (Dai and Yin, 2018), which is expressed using the following equation.


(4) $$Y_{i,SNV}=\left(Y_{i,k}-Y_{i}\right)/\sqrt{\sum_{k=1}^{n}{\left(Y_{i,k}-Y_{i}\right)}^{2}/(n-1)}$$

where *Y_i,SNV_* is the spectral matrix transformed by the standard normal variable; *Y_i,k_* is the reflectance value of the *i*th sample in the *k* th band; *Y_i_* is the mean value of the hyperspectral emissivity of the *i*th sample, and *n* is the total number of bands.

### 2.4 Spectral band selection

Despite its fine spectral information, UAV hyperspectrum is prone to problems such as dimensionality disaster, overfitting, and reduced characterization due to its multi-spectral bands, large amount of data, redundant information, and high complexity. To solve this problem, it is imperative to select the premium hyperspectral bands, choosing the main subsets from the original hyperspectral bands to reduce data dimensionality while retaining the useful information in a relatively complete manner. According to an analysis of various band optimization algorithms, correlation analysis, SPA, OIF, and BCI were used to select the optimal hyperspectral bands.

#### 2.4.1 SPA

SPA is a forward-iterative search algorithm that minimizes the collinearity of the vector space (Araújo et al., 2001) and whose basic principle is as follows. Firstly, the spectral matrix *X*_*M*×*K*_ is constructed, with *M* as the number of samples, *K* as the number of bands, ***x*_k_**_(0)_ as the initial iteration vector, and *N* as the number of wavelengths to be extracted. Then, starting from one wavelength, this wavelength is projected to others, where in each cycle, the projection vectors are compared, and the wavelength with the largest projection vector is stored in the set of wavelengths to be selected from; this is repeated for *N* (the number of wavelengths to be extracted) cycles. The wavelength stored each time has the smallest amount of redundant information and collinearity with the previous wavelength. Finally, multiple linear regression (MLR) is established for the wavelength combinations obtained from different ***x*_k_**_(0)_ and *N*, and it is analyzed using RMSE and leave-one-out cross-validation (LOOCV) of the modeling set. The band combination with the minimum RMSE value is selected as the optimal band (Zhang et al., 2021).

#### 2.4.2 OIF

OIF, first proposed by Chavez et al. (Chavez et al., 1982), is the most widely used spectral band optimization method. It has the underlying principle that the greater the ratio of the sum of the standard deviations of the bands to the sum of the correlation coefficients of the combined bands, the greater the amount of information contained in the band combination, thereby the lesser the redundant information. Its expression is as follows.


(5) $$\text{OIF}=\sum_{i=1}^{n}S_{i}/\sum_{i=1}^{n}\left|R_{ij}\right|$$

where *S_i_ S*_*i*_ is the standard deviation of the *i* th band, and *R_ij_* is the correlation coefficient between the *i* th and the *j* th band.

#### 2.4.3 BCI

The basic principle of BCI is to combine the spectra at any two bands and to analyze the linear correlation between the combined spectral indexes and the monitoring index to achieve the optimal selection of spectral bands by comparing the correlation coefficients (Tang et al., 2021).

### 2.5 Spectral index construction

In order to examine the information contained in the spectral data and reduce the influence of soil background and of atmospheric and radiation errors on the spectral data, the NDSI, RSI, and SSI were constructed, as expressed by the formulas below.


(6) $${\text{NDSI}}_{\left(\lambda_{1},\lambda_{2}\right)}=\left(R_{\lambda_{1}}-R_{\lambda_{2}}\right)/\left(R_{\lambda_{1}}+R_{\lambda_{2}}\right)$$

(7) $${\text{RSI}}_{\left(\lambda_{1},\lambda_{2}\right)}=R_{\lambda_{1}}/R_{\lambda_{2,}}$$

(8) $${\text{SSI}}_{\left(\lambda_{1},\lambda_{2}\right)}=R_{\lambda_{1}}-R_{\lambda_{2,}}$$

where *R*_*λ*_1__ is the canopy reflectance at the wavelength of *λ*_1_ , and *R*_*λ*_2__ is the canopy reflectance at wavelength *λ*_2_. MATLAB 2021b was used to calculate the spectral reflectance, and NDSI, RSI, and SSI were obtained.

### 2.6 PLSR

PLSR is a regression method that combines principal component analysis (PCA) and multiple linear stepwise regression (MLSR) (Uk et al., 2022). Owing to its advantages such as data dimensionality reduction, information synthesis and screening, elimination of redundancy and collinearity, PLSR is widely used in spectral data processing. Its basic principle is to extract the independent *T*_*h*_(*h*=1,2,···) and *U*_*h*_(*h*=1,2,···) dependent variable sums from independent variables X(*x*_1_,*x*_2_,···,*x*_*n*_) and dependent variables Y(*y*_1_,*y*_2_,···,*y*_*n*_) through PCA, and maximize the covariance between the sums of *T*_*h*_ and *U*_*h*_ to establish an MLSR model as expressed below:


(9) $$\text{X=}T_{h}{\text{P}}^{T}+\text{E}$$

(10) $$\text{Y=}U_{h}{\text{Q}}^{T}+\text{F}$$

Where P and Q are the orthogonal load matrix of *n*×*h* ; E and F are the errors that conform to the normal distribution.

### 2.7 Analysis and evaluation

To analyze the accuracy of the CGI characterization model, the coefficient of determination *R* ^2^ , RMSE, and RE were used to evaluate the results. The larger the value of *R* ^2^ , the smaller the RMSE and the better the prediction performance and accuracy of the constructed model. When RE≤10% , the model is premium; when 10*%*<RE≤20% , the model is moderate; the model is poor when RE>20% (Wang et al., 2022), as expressed below.


(11) $$R^{2}=1-\sum_{i=1}^{n}{\left(y_{i}-{\hat{y}}_{i}\right)}^{2}/\sum_{i=1}^{n}{\left(y_{i}-{\bar{y}}_{i}\right)}^{2}$$

(12) $$\text{RMSE}=\sqrt{\frac{1}{n}\sum_{i=1}^{n}{\left(y_{i}-{\hat{y}}_{i}\right)}^{2}}$$

(13) $$\text{RE}=\text{RMSE}/{\bar{y}}_{i}\times 100\%$$

where *n* is the sample size; *y*_*i*_ is the predicted value; ${\hat{y}}_{i}$ is the actual measurement value, and ${\bar{y}}_{i}$ is the mean actual measurement.

## 3 Results and analysis

### 3.1 Statistical result analysis of indicators in the study area

**Figure 4:**
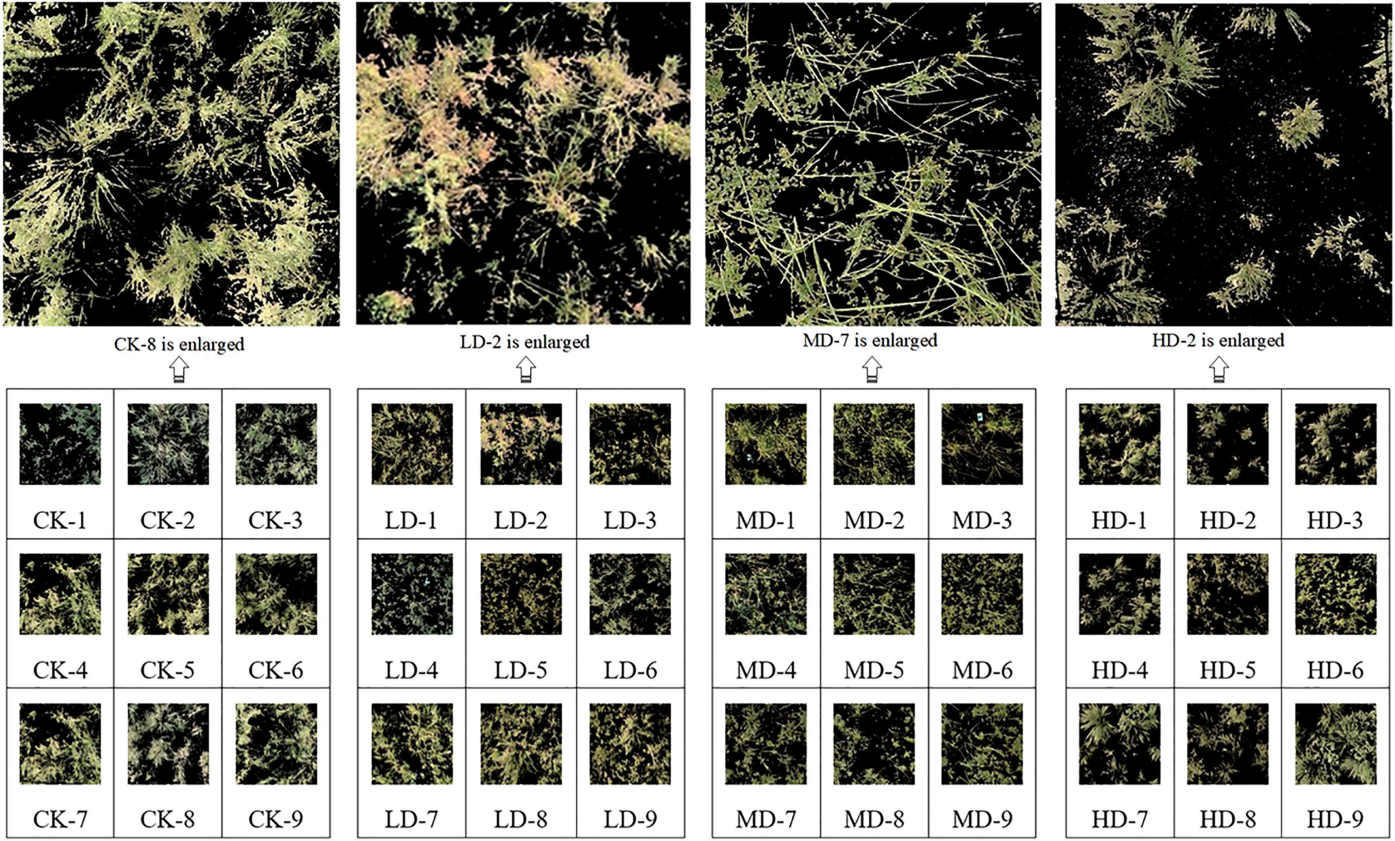
Quadrat image segmentation results.

An example of the segmentation and processing results for the compensated images using K-means clustering algorithm is illustrated in **Figure 4**. The latitudes and longitudes, vegetation coverage, AGB, moisture content, species richness, and community height of the four study areas are presented in **Table 1**, which shows that each statistical indicator exhibits a decreasing trend with increasing grassland degradation gradient. Thus, the selection of the sample plot and the division of the degradation gradients are accurate and reasonable.

**Table 1:** Community characteristics of experimental plots.

Point of sampling	Latitude /^o^N	Longitude /^o^E	FVC /%	AGB /g•m^-2^	Moisture content /%	Species richness/n	Average community height /cm	CGI
CK	39°19′33.51″~ 39°19′49.67″	107°91′77.66″~ 107°91′94.52″	40.07	146.55	42.59	9.67	24.73	0.5577
LD	39°18′07.75″~ 39°18′21.09″	107°91′16.91″~ 107°91′33.56″	34.86	122.66	39.83	6.22	17.55	0.5075
MD	39°17′08.19″~ 39°17′20.95″	107°87′97.07″~ 107°88′14.58″	30.58	102.96	37.54	5.33	10.24	0.4529
HD	39°17′89.90″~ 39°18′03.04″	107°24′34.91″~ 107°24′51.26″	29.05	87.51	32.41	3.33	12.10	0.4106

The number of quadrats in each experimental area was 9; The undegraded area (CK), light degradation area (LD), moderate degradation area (MD), and high degradation area (HD); fractional vegetation cover (FVC), above ground biomass (AGB), comprehensive growth index (CGI).

### 3.2 Analysis of spectral data preprocessing and band optimization results

#### 3.2.1 Analysis of spectral data preprocessing results

**Figure 5:**
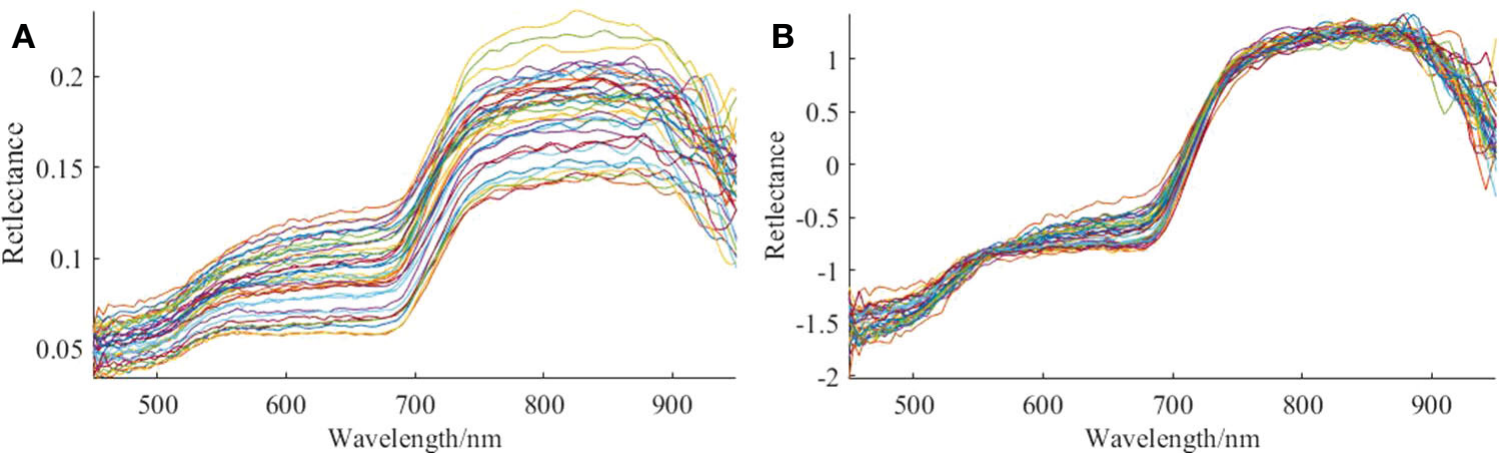
Hyperspectral preprocessing results. **(A)** Average reflectance spectra of canopy **(B)**. Spectrum diagram of SNV algorithm preprocessing.

The spectral data were processed using SNV, and the preprocessing results of the 36-quadrat canopy hyperspectral data are illustrated in **Figure 5**, with **Figure 5A** being the original spectrogram, and **Figure 5B** being the spectrogram after preprocessing. It can be observed from **Figure 5B** that using SNV to preprocess the original spectrum effectively eliminates the interference of noise and surface scattering. The reflectance changes as the reflectance curves in the 450–530 nm cyan-blue and 850–950 nm near-infrared spectra become more dispersed, and those in the 530–850 nm yellow-green, orange, and red spectra become more concentrated.

#### 3.2.2 Analysis of spectral band optimization results

(1) Spectra Screened by the Correlation Analysis

**Figure 6:**
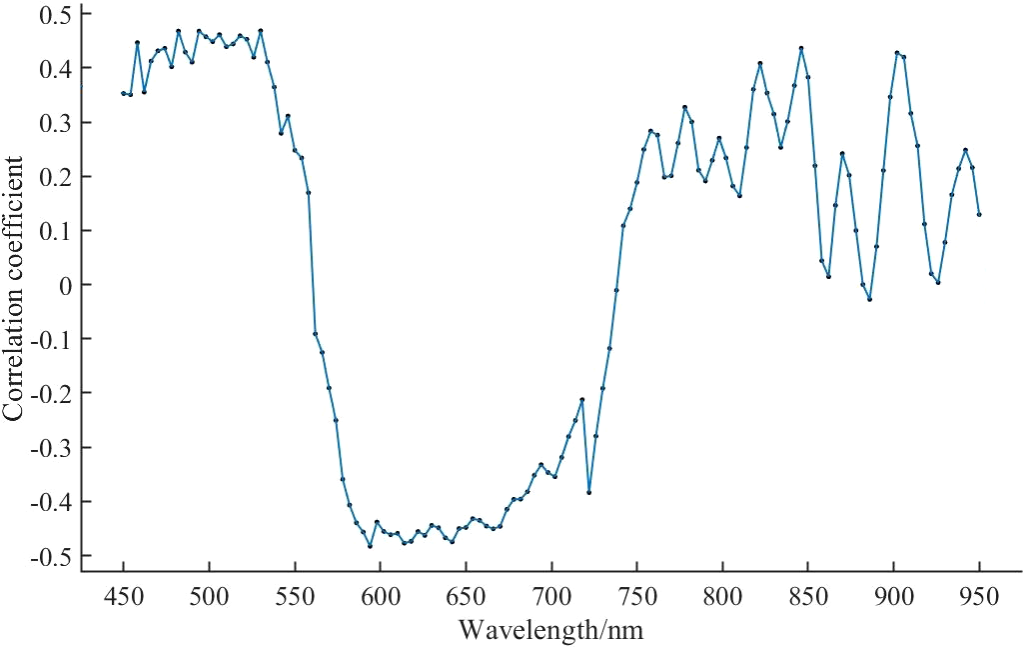
Correlation analysis of spectra and CGI.

The correlation analysis was performed between the pre-processed spectral curves and the CGI. The correlation coefficients between the spectra and CGI were calculated; the results are illustrated in **Figure 6**, and the characteristic bands are presented in **Table 2**. The results show that the spectral reflectance of the pre-processed spectra in the 450–558 nm and 742–950 nm bands is positively correlated with CGI, with a maximum positive correlation coefficient of approximately 0.4686, located at 530 nm. The spectral reflectance in the 562–950 nm band is negatively correlated with CGI, with a maximum negative correlation coefficient of approximately -0.4829, located at 594 nm.

**Table 2:** Characteristic band and maximum correlation coefficient.

	Main sensitive band/nm	Maximum positive correlation coefficient	Maximum negative correlation coefficient
Hyperspectral pretreatment by SNV method	458、466~534; 582~674	0.4686	-0.4829

(2) Spectra Screened by the SPA

**Figure 7:**
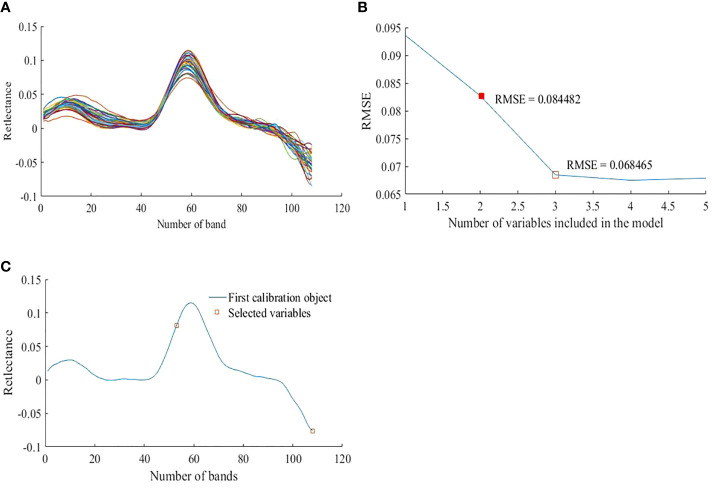
Spectra Screened by the SPA. **(A)** Spectrum diagram of Savitzky-Golay smoothing **(B)** The curve of root mean square error (n = 24) **(C)**. Characteristics variable selection results.

The processing results of the preprocessed 36 quadrature vegetation canopy spectral data in SPA are illustrated in **Figure 7**, where 24 groups are training samples, and 12 groups are validation samples. **Figure 7A** shows the first-order result of the Savitzky–Golay smoothing, and the spectral curve after the first-order smoothing is more concentrated. The relationship between RMSE in the modeling set LOOCV and the number of characteristic bands is illustrated in **Figure 7B**. The boxes indicate the positions of the marked characteristic bands. RMSE decreases rapidly with the increase in the number of characteristic bands. When the number of characteristic bands was 3, the RMSE reached a minimum value of 0.068465. However, to facilitate the subsequent construction of the dual-band spectral index and improve the computational speed, the number of characteristic bands was set to 2. At this point, the RMSE value was 0.084482, which is a difference of 0.016017 from the RMSE value when the number of characteristic bands was 3. Therefore, two characteristic bands were selected as the best band combination. **Figure 7C** illustrates the results of the selection of characteristic bands. The red squares indicate the positions of the characteristic bands (53 and 108) screened out by the SPA algorithm after preprocessing, and the wavelength combination is 658 nm and 878 nm, distributed in the red light range and the near-infrared spectral range, respectively.

(3) Spectra Screened by the OIF

The number of band combinations obtained from OIF was larger. Only the first five OIF values are listed in **Table 3**, showing that the OIF value of the wavelength combination of 450 nm and 942 nm is the highest (0.1033). The correlation coefficient is the lowest at 0.2884, which is significantly lower than that of the other four band combinations, and the bands are distributed in the cyan and near-infrared spectral regions.

**Table 3:** Band combinations obtained from OIF (n=24).

Sequence	Band Combination	Optimal OIF value	Standard deviation	Correlation coefficient
1	R_450_—R_942_	0.1033	0.0298	0.2884
2	R_666_—R_950_	0.0749	0.0443	0.5915
3	R_502_—R_950_	0.0737	0.0350	0.4752
4	R_630_—R_950_	0.0718	0.0436	0.6075
5	R_926_—R_950_	0.0632	0.0464	0.7337

R is spectral reflectance.

(4) Spectra Screened by the BCI

**Figure 8:**
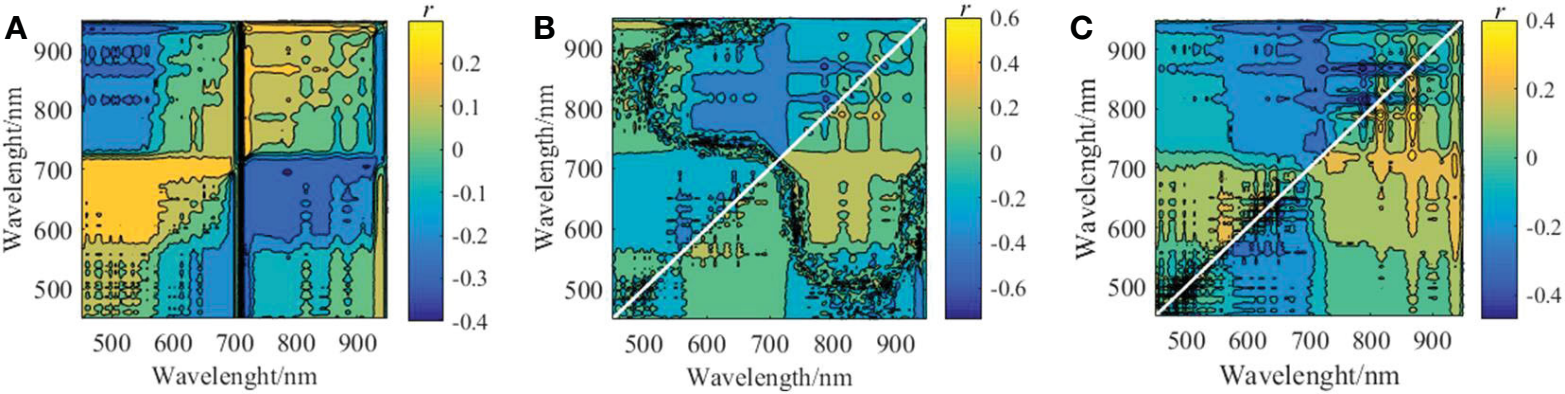
Correlation coefficients between spectral indices and CGI (n = 24) **(A)**. *r* for RSI and CGI **(B)**. *r* for NDSI and CG **(C)**. *r* for SSI and CGI.

The RSI, NDSI, and SSI spectral indices of the combination of two arbitrary bands in the hyperspectral data from the 126 bands in 24 training samples were constructed using BCI. The correlation coefficients are illustrated in **Figure 8**, where the correlation coefficient *r* of RSI_(478,710)_ is 0.3973, the correlation coefficient *r* of NDSI_(714,710)_ is 0.73958, and the correlation coefficient *r* of SSI_(650,646)_ is 0.47052, NDSI_(714,710)_ and SSI_(650,646)_ are composed of the spectrum in the red light region, RSI_(478,710)_ consists of the spectrum in the red and cyan light regions, and correlation coefficient *r* highest value of NDSI_(714,710)_ , which is mainly caused by the sensitivity of the spectrum in the red light region to green plants and its linear relationship with CGI, showing a high fitting accuracy.

### 3.3 Result analysis of grassland vegetation CGI characterization model

In the random sampling of the data from the 36 quadrats, with 24 groups of data as training samples and 12 groups of data as validation samples, Several spectral indices were taken as input variables: Original spectra; The optimal spectra screened by the correlation analysis; RSI (658,878), NDSI (658,878), and SSI (658,878), corresponding to the optimal spectra screened by the SPA method; RSI (450,942), NDSI (450,942), and SSI (450,942), corresponding to the optimal spectra screened by the OIF method; RSI (710,478), NDSI (714,710), and SSI (650,646), corresponding to the optimal spectra screened by the BCI method, respectively. The CGI of grassland vegetation was taken as the dependent variable, respectively. The accuracy of the CGI of grassland vegetation characterization model constructed with the PLSR algorithm is presented in **Table 4**. The following can be observed from **Table 4**: (1) *R* ^2^ was greater than 0.6 for models built using the spectral indices corresponding to the optimal spectra screened by the SPA method and the spectral indices corresponding to the optimal spectra screened by the BCI method; this result indicates significant modeling accuracy improvement compared with other indices. (2) The *R* ^2^ of models built using the training samples of the original full-band spectra was 0.4621, RMSE was 0.1034, and RE was 12.01%, indicating significant modeling accuracy improvement after the screening of optimal spectra. Moreover, the model computation was significantly reduced, and the validation samples were highly correlated. (3) The *R* ^2^ of models built using the training samples of the spectral indices corresponding to the optimal spectra screened by the SPA method was 0.7835, RMSE was 0.0712, and RE was 6.89%, less than 10%. The *R* ^2^ of the Validation samples was 0.7698, RMSE was 0.0471, and RE was 6.36%, less than 10%, highest precision.

**Table 4:** Precision evaluation of CGI estimation based on PLSR algorithm.

Modeling index	Principal factor number	Training samples			Validation samples		
		*R* ^2^	RMSE	RE(%)	*R* ^2^	RMSE	RE(%)
Original spectra	5	0.4621	0.1034	12.01	0.4199	0.1216	15.21
Optimal spectra screened by the correlation analysis	2	0.5510	0.0882	9.67	0.5244	0.1202	11.11
RSI (658,878), NDSI (658,878), and SSI (658,878), corresponding to the optimal spectra screened by the SPA method	2	0.7835	0.0712	6.89	0.7698	0.0471	6.36
RSI (450,942), NDSI (450,942), and SSI (450,942), corresponding to the optimal spectra screened by the OIF method	2	0.4936	0.0905	17.76	0.4988	0.0663	17.58
RSI (710,478), NDSI (714,710), and SSI (650,646), corresponding to the optimal spectra screened by the BCI method	2	0.637	0.0812	7.98	0.699	0.0637	7.46

**Figure 9:**
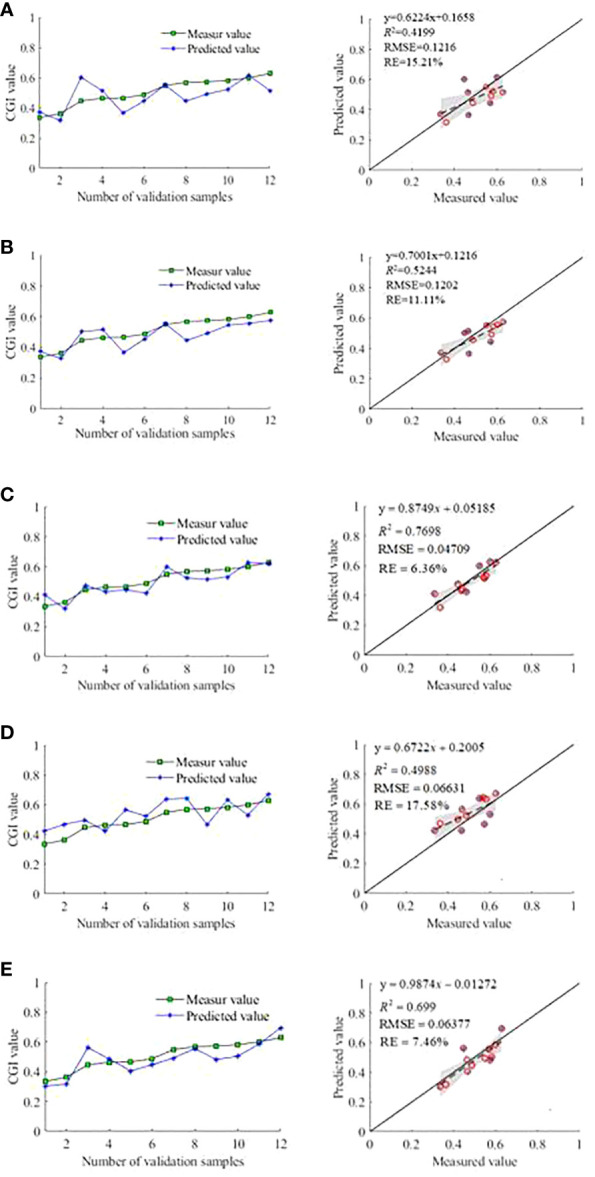
Accuracy validation of each modeling method. **(A)** Inverse CGI accuracy verification of models built using the original spectra **(B)**. Inverse CGI accuracy verification of models built using the optimal spectra screened by the correlation analysis **(C)**. Inverse CGI accuracy verification of models built using the optimal spectra screened by the SPA method **(D)** Inverse CGI accuracy verification of models built using the optimal spectra screened by the OIF method **(E)**. Inverse CGI accuracy verification of models built using the optimal spectra screened by the BCI method.

In the application of grassland vegetation characterization model constructed with the PLSR algorithm to characterize the 12 sets of validation samples, the confidence interval was set at 95%, and the results are shown in **Figure 9**. It can be seen that the models built using the spectral indices corresponding to the optimal spectra screened by the SPA method has the highest accuracy.

### 3.4 Grassland vegetation CGI monitoring and result analysis

**Figure 10:**
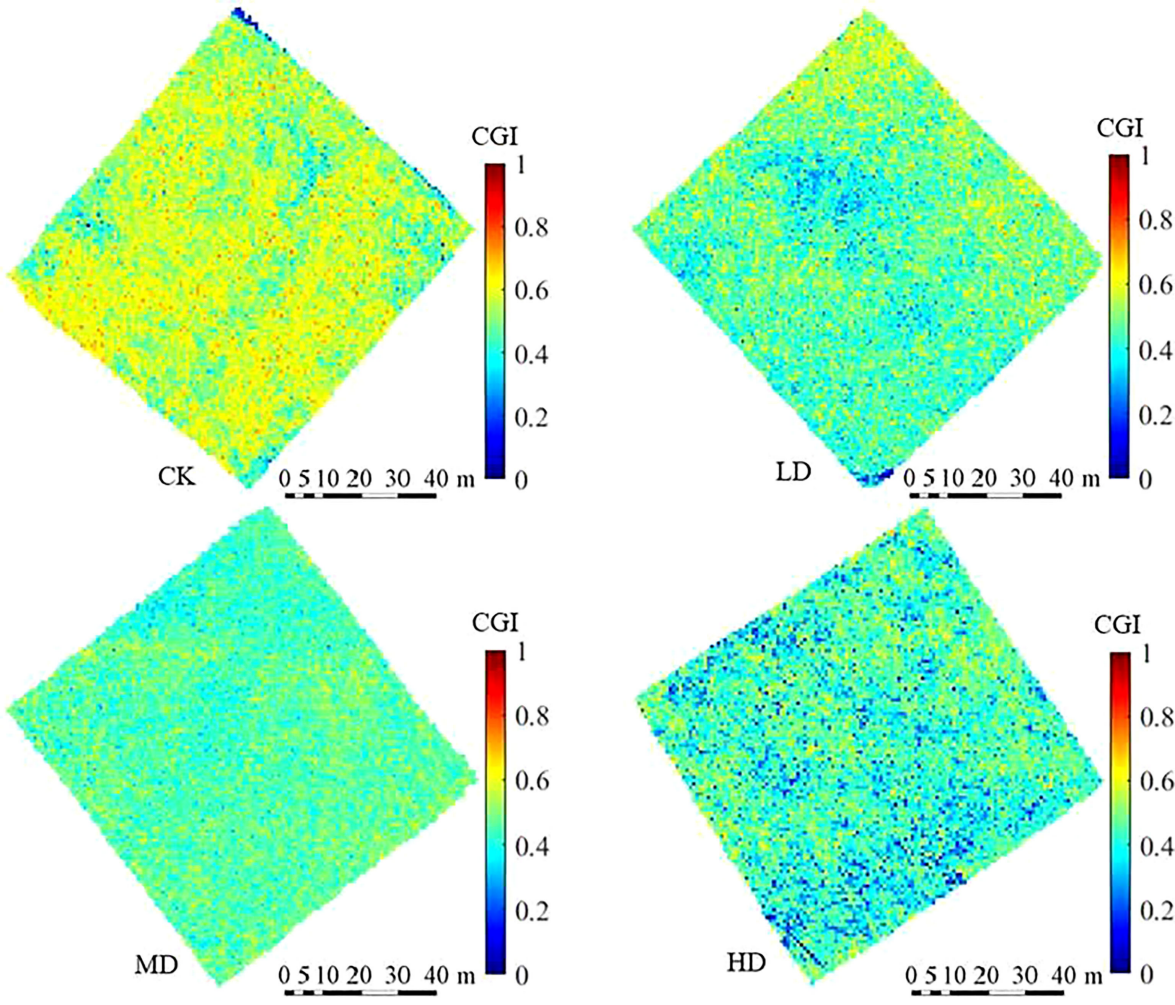
Inversion results of CGI grassland vegetation in four study areas.

The models built using the optimal spectra screened by the SPA method was used to calculate the CGI of grassland vegetation on a pixel-by-pixel basis in the four study areas. The results were mapped and are illustrated in **Figure 10**. It can be seen from the figure that the color of the CGI distribution map gradually turns blue as the degree of grassland degradation intensifies, i.e., the CGI value gradually decreases, which is consistent with the actual situation. The grassland vegetation growth in the study area can be clearly identified in the CGI distribution map.

**Table 5** presents the comparative results between the inversion CGI mean value of the SPA_PLSR model and that of the quadrats. It can be seen from the table that the mean CGI values of the quadrats in areas CK, LD, MD, and HD were 0.5577, 0.5075, 0.4529, and 0.4106, respectively, and the mean CGI values of the SPA_PLSR model inversion were 0.0.5606, 0.4735, 0.4659, and 0.4319, respectively, i.e., an average RE of 3.82%. Therefore, the model can adequately monitor the vegetation growth of desert steppe grasslands.

**Table 5:** Comparison of model prediction and quadratic mean CGI results.

	CK CGI	LD CGI	MD CGI	HD CGI	Relative mean/%
Quadratic mean	0.5577	0.5075	0.4529	0.4106	3.82
Model prediction mean	0.5606	0.4735	0.4659	0.4319	

## 4 Discussion

Under consistent regional background conditions, The quality of regional ecology is mainly determined by the characteristic index features of vegetation. From the perspective of ecological characteristics, the ecological dynamic changes of desert steppe are mainly manifested through indicators such as FVC, SR, ACH, AGB, and VMC. The FVC, the proportion of dominant species and SR are the main factors that reflect community difference and structure. Under accurately selecting monitoring indices is critical for the monitoring of grassland conditions and ecological quality assessment studies, as single-index monitoring often produces bias or errors. Therefore, a new index, namely the CGI, was established in this study by integrating the indices reflecting the growth status of grassland vegetation. Inverse models of the CGI of grassland vegetation with several spectral indices were constructed using UAV hyperspectral data of grasslands with varying degrees of degradation. Satisfactory results were obtained by applying the models. It can accurately characterize of vegetation indexs in desert steppe with a low vegetation cover, low plant height, and high soil brightness.

Comparative analyses showed that the inversion accuracy of models built using the optimal screened spectra was higher than that of using the original spectra. This was mainly because the original spectra contained high and significant band redundancy, and invalid spectra affected the modeling results. Therefore, it was necessary to screen the optimal hyperspectral band, which is consistent with previous studies (Zhang et al., 2017; Yu et al., 2022). The optimal spectra were screened using correlation analysis, and the modeling accuracy of sensitive screened bands was improved. However, the effect did not meet demands, which was mainly because the FVC of desert-steppe grasslands is low, vegetation is low, and the soil brightness is high. These factors easily mask the spectral contribution of vegetation in the image pixel. The modeling accuracy of spectral indices was better than that of original spectra and that of models built using the optimal spectra screened by correlation analysis. The accuracy of models built using optimal spectra screened by the SPA method was the highest. This was mainly because the spectral index was derived by mathematical combination operations of several band data, which not only yielded better sensitivity than the one-dimensional spectrum, but also better eliminated the intra-band autocorrelation. Consequently, the environmental noise was reduced or eliminated, the spectral feature response was enhanced, and the modeling accuracy was improved.

In this study, the hyperspectral bands are optimized with SPA, OIF, and BCI respectively, and the sensitive bands corresponding to the optimal NDSI spectral index are basically in the near-infrared band and the red band. This is a comprehensive reflection of the vegetation type, coverage, and growth status, etc. in good correlation with CGI, which is consistent with the research results of many scholars (Sebastian et al., 2015; Liu et al., 2018). This study constructed a multi-spectral index CGI inversion model using UAV hyperspectral data having different degrees of degradation and achieved satisfactory results. However, there are still shortcomings in this study. 1) The research area for this study was mainly in the desert steppe in Otuoke Banner, Ordos, hence the relevance and universality of the findings need to be verified, and subsequent experiments need to be carried out on desert steppes in other regions or even other types of grasslands to test the universality of the model. 2) In this study, the influence of soil background and litter on the spectral reflectance is not considered in depth. In addition, the influence of sensor observation angle and solar elevation angle on the inversion model demands further investigation efforts.

## 5 Conclusion

This study examined the CGI monitoring of grassland vegetation and investigated the applicability of UAV hyperspectral data for analyzing the CGI characterization of desert-steppe grassland vegetation. We conducted a correlation analysis between pre-processed spectral curves with the CGI and extracted sensitive bands at 458 nm, 466–534 nm, and 582–674 nm. The optimal spectral indices screened by SPA were RSI (658,878), NDSI (658,878), and SSI (658,878); those selected by OIF were RSI (450,942), NDSI (450,942), and SSI (450,942); those by BCI were RSI (710,478), NDSI (714,710), and SSI (650,646). Models were built using the spectral indices corresponding to the optimal spectra screened by the SPA method, and the CGI mean values were inverted. A comparison of the mean measured CGI values of the sample quadrat of the test area showed that the mean relative error was 3.82%, this model has the highest accuracy. These results show that UAV hyperspectral remote sensing can accurately monitor the CGI of grassland vegetation, providing an effective method to quickly obtain information on grassland conditions.

## Data availability statement

The original contributions presented in the study are included in the article/supplementary material. Further inquiries can be directed to the corresponding author.

## Author contributions

XL and ZP conceived and designed the experiments. YC, YY, XS, KS, YL and JZ performed the experiments. HW analyzed the data and wrote the paper. All authors contributed to the article and approved the submitted version.
